# Pilot implementation of a routine immunization module of the district health information system version 2 in Kano State, Nigeria, 2014 - 2015

**DOI:** 10.11604/pamj.supp.2021.40.1.24879

**Published:** 2021-11-12

**Authors:** Belinda Vernyuy Uba, Ndadilnasiya Endie Waziri, Oluwasegun Joel Adegoke, Adekunle Akerele, Saheed Gidado, Nnamdi Usifoh, Olorunsogo Bidemi Adeoye, Charles Micheal Akataobi, Suleiman Haladu Ahmed, Ramatu Usman Obansa, Edwin Simple, Beza Kibret, Chima Ohuabunwo, Oladayo Biya, Eric Wiesen, Chime Nnadi, Patrick Nguku

**Affiliations:** 1 African Field Epidemiology Network, Abuja, Nigeria,; 2 United States Centre for Disease Control and Prevention, Atlanta, Georgia

**Keywords:** Routine immunization, District Health Information System, Nigeria, reporting

## Abstract

**Introduction:**

Timely and accurate data are necessary for informing sound decision-making and developing effective routine immunization (RI) programs. We launched a pilot project in Kano State to strengthen routine immunization (RI) data reporting through the immunization module of the District Health Information System version 2 (DHIS2). We examined the completeness and timeliness of reporting monthly RI data one year before and one year after DHIS2 module pilot in the State.

**Methods:**

The first phase of the DHIS2 RI module pilot in Kano included training on RI data tools in November 2014 and in January 2015 for 36 state and zonal personnels, 276 local government area (LGA) personnel, and 2,423 health facility (HF) staff. A RI-focused dashboard to display core RI accountability framework indicators, such as completeness and timeliness of reporting, planned immunization sessions conducted, coverage and dropout was implemented. Report completeness was ratio of submitted reports to number of health facilities while report timeliness was ratio of reports on the DHIS2 by 14th of the month to number of expected.

**Results:**

Completeness of data reporting increase from 70% in 2014 to 87% in 2015, while timeliness of reporting increase from 64% to 87% over the same period. Challenges encountered during the implementation process included limited access to internet, power outages, health workers strike, staff attrition and competing state activities.

**Conclusion:**

The pilot implementation of the DHIS2 immunization module in Kano State led to modest improvement in the reporting of RI services. Several lessons learned were used to guide scale-up to other states in the country.

## Introduction

Timely and high-quality routine immunization (RI) data are necessary to inform sound decision-making at all levels of program and policy in order to develop and manage effective RI programs [1]. A robust system for collecting, reporting and archiving RI data generates accurate and timely information for effective planning, monitoring and evaluation of RI service delivery, contributing to increased efficiency in the utilization of resources and improved program outcomes [1, 2].

Whilst a trend toward electronic data collection appears to be gaining momentum globally [1], Nigeria until recently had a system dominated by paper-based data collection and storage tools; chronic stock-outs of these tools and their effects on data reporting have been documented [3]. Manual data aggregation at multiple levels contribute to deficiencies in data accuracy, completeness, timeliness and reliability of reporting that adversely affect program planning and service delivery [1-3]. The RI delivery system suffers from inadequate manpower relative to need and high staff turnover at the health facility (HF) level. While the capacity of health workers appears to be improving at all program levels [4], recruitment and retention of trained RI workers in rural HFs remain a major challenge [4]. When available, these workers report heavy workloads that negatively affect attentiveness to data collection and reporting. High staff attrition rates and lack of regular feedback and supervision have been linked with problems of data quality and use [4, 5]. Insufficient state and Local Government Area (LGA) government ownership of strengthening RI delivery and data systems [6, 7] has resulted in inadequacies in RI data management and program implementation as indicated by surveys finding lower coverage rates compared to those reported by the administrative monitoring system [4, 5].

Two RI data systems have existed in Nigeria: the Nigerian government-owned National Health Information Management System (NHMIS), initiated in 2006 and the WHO-administered District Vaccine Data Management Tool (DVD-MT), a Microsoft Excel-based reporting tool in use since the inception of EPI in Nigeria in 1979 to manage vaccine stocks and monitor administrative vaccination coverage. However, the former lacked several critical elements for easy and accurate tracking of immunization programm performance. The District Health Information System version 2 (DHIS2) platform used for NHMIS with additional routine immunization indicators was meant to improve data availability, visibility and strengthen the monitoring of RI indicators and data use for action at all levels and replace the DVD-MT.

On the DVD-MT, RI data are aggregated at each level: HFs collect data on the number of vaccine doses administered during a one-month period. The summary report is sent to LGA public health authorities who are to review the data and take necessary actions, such as following-up on non-reporting facilities, investigating unusual coverage data, and conducting data quality checks [8]. The aggregated data for each LGA are submitted to the state from where it is further summarized and transmitted to the national levels for compilation, analysis and used for decision making, resource allocation, and integrated support from partners [9]. The multiple levels of data collation and person-to-person reporting; at the LGA, state and national provided opportunities for errors and poor data quality.

The DHIS software is a software that integrates all health data sources into a single integrated health information system. The DHIS2 RI module provides access to real time data on a dedicated RI dashboard that makes available RI data disaggregated from States, LGAs, wards, down to the health facility level for easy verification. It provides a comprehensive platform for managing information on the logistics and administration of vaccinations for decision making by government agencies and stakeholders thus creating opportunity for improving the overall immunization data system. It has a platform for data entry, analysis and display of a dashboard that contains all the RI indicators as required on the Accountability Framework for RI in Nigeria (AFRIIN).

The Nigeria Federal Ministry of Health (FMoH) adopted and customized DHIS version 1.4 in 2006 as the national HMIS platform. Subsequently, the updated version DHIS2 was adopted in 2011. This platform is a highly flexible tool for health data management and integration. Information on the DHIS2 platform can be accessed from any location with an internet connection, and it allows health professionals to access real-time information for decision making [10, 11]. To integrate all RI data into this platform, an RI module was added to the Nigerian DHIS2 in 2014 by the National Primary Health Care Development Agency (NPHCDA) in collaboration with the FMoH. This module was intended to manage and monitor information on the distribution and administration of vaccine doses and key RI indicators. Accountability framework indicators, such as the proportion of planned immunization sessions (fixed/outreach) that were conducted, vaccine stock management, funds availability, cold chain functionality, monthly/annual administrative coverage and dropout would be available for analysis, and an RI dashboard could efficiently monitor these indicators.

Kano State has 44 LGAs. With an estimated population 9,401,288 in the 2006 census, the projected population in 2015 was 12,652,397 of which 506,213 were younger than 12 months [12]. The DHIS2 RI module was piloted in Kano State to learn lessons prior to plan nationwide scale-up. The objectives of implementing DHIS2 RI module were to 1) enhance and institutionalize the use of the DHIS2 RI dashboard to visualize RI indicators and increase the use of quality RI data for decision-making at state, LGA, and HF levels, 2) monitor key indicators of timeliness and completeness of reporting and antigen coverage rates, and 3) ensure data is available for use during supportive supervision. This paper outlines our experience and lessons learned from the implementation of the DHIS2 RI module in Kano State.

## Methods

The DHIS2 immunization module was progressively implemented in Kano State from November 2014 with routine monitoring of RI indicators. The critical elements of the project included a needs assessment to determine capacity of personnel and resources available for its implementation, modification of existing national RI data tools, development and testing of an RI-focused dashboard, DHIS2 trainings, introduction of standard operating procedures for systematic Data Quality Use and Supportive Supervision (DQUSS), and implementation of DHIS2 Core Group and review meetings to enhance data quality and programmatic use. For this pilot, the impact of DHIS2 RI module implementation was primarily measured by the completeness and timeliness of monthly RI data reporting. Data reporting completeness was measured by the proportion of monthly LGA data submitted to the state level out of all expected monthly reports. Timeliness was measured by the proportion of monthly LGA data submitted by the requested date of all expected monthly reports.

### Needs assessment

In this phase a tool was developed to gather the state´s requirements to implementing DHIS2 RI module successfully. Using the tool, a needs assessment to identify areas of strengths and weaknesses in the existing system was conducted. Key informant interviews were conducted with the leadership of the state agency while a questionnaire administered to health workers in selected LGAs and health facilities. Specifically, the assessment examined: 1) staffing needs for DHIS2 module use 2) gaps in capacity to utilize the DHIS2 platform at LGA and filling data tools at health facilities 3) RI data tool availability for routine monthly data reporting from HFs to the LGA for entry into the DHIS2 platform.

### Modification of RI data collection tools

Modifications were made across all RI data collection tools used at the HF, LGA and state levels to capture additional data elements that were hitherto not captured on the national data tools in line with the accountability framework for RI indicators required for the DHIS2 RI dashboard. These modifications included insertion of new data elements to all existing RI data tools including those elements for monitoring RI indicators and creation of tools for collecting HF vaccine utilization data. The revised data tools were rolled out during the implementation of the DHIS2 module in the State.

### Development and testing of an RI module

The FMoH and NPHCDA with technical support from the Nigeria Health Information System Program (HISP), and other partners, led the development of the customized RI dashboard and conducted a test run of the module. The DHIS2 RI module was initially on a test server for training and pilot purposes. Subsequently, the DHIS2 RI module was migrated from a test server to the national instance managed by the FMoH.

### Trainings

The training module was designed based on findings from the needs assessment. Content of the training included the overview of the revised RI data tools (one-day session) and overview of the DHIS2 RI module (three-day session) with hands-on practice on filling out data tools, transcription of data from the old tools to the revised monthly vaccine summary form, data entry into the DHIS2 platform, data analysis using the pivot table tool on the platform and review of the RI dashboard indicators. Due to competing activities in the state, and to ensure efficiency in facilitators to participants ratio the training was conducted in two phases, in November 2014 and January 2015with 24 LGA in phase 1 and 20 LGA in phase 2.

The training was conducted at two levels: state level and LGA level. Participants at the state level included local immunization officers, cold chain officers, supportive supervision officers and the monitoring and evaluation officers. For the LGA level training, the participants at the state level training were deployed to their LGAs to cascade the “how to fill RI data tool part of the training”. Participants at the LGA level training consisted of the RI focal person from each health facility and the assistant or a records officer from the health facility.

### DHIS2 core group and joint review meetings

**Figure 1:**
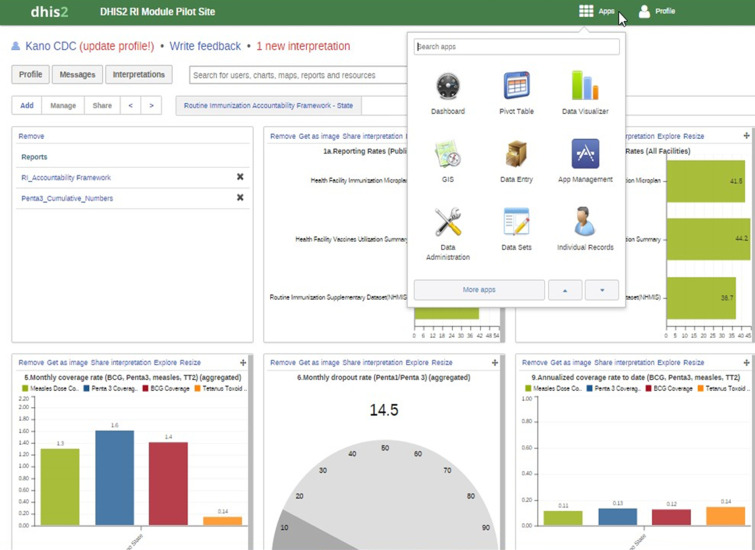
Routine immunization Dashboard on the DHIS2 Platform, Kano State Nigeria, 2014

To enhance and sustain the accuracy of RI data for decision making, a state-level DHIS2 Core Group was established. The group was comprised of Monitoring and Evaluation (M&E) Officers for immunization under the SPHCDA, the agency Director, key officers overseeing the different components of RI service delivery and representatives of partner agencies supporting the EPI program. This group was tasked to review the DHIS2 dashboard on a monthly basis to monitor the utilization of DHIS2 at the LGAs and assess RI indicators. They were expected to monitor and review data, provide feedback, and strategically use the data to make informed decisions based on the RI indicators that are displayed on the dashboard as shown in Figure 1.

Under-reporting LGAs and those with data quality issues (e.g., detection of data entry errors) were identified; follow-up supervision was conducted to address these problems. A set of validation rules were set and tracked on the platform to detect discordance in different variables of LGA data in the module. A decrease in the number of LGAs with discordant entries was used as a proxy for improvement in data quality. The DHIS2 Core Group was transformed in November 2015 to an M&E Working Group with the addition of M&E Officers from all other programs of the SPHCDA and the FMoH HMIS Officer. Kano State officials supported joint meetings of M&E Officers and LIOs to review RI data for progress toward improvement of data completeness, timeliness and accuracy as well as of program implementation.

## Results

A total of 2,735 participants were trained across phase 1 and phase 2. The state level training consisted of 220 participants (local immunization officers (44), Cold Chain Officer (44), monitoring and evaluation officers (44) and supportive supervision officers (88)). The LGA level training participants were 2515 (2 participant per health facility).

**Figure 2:**
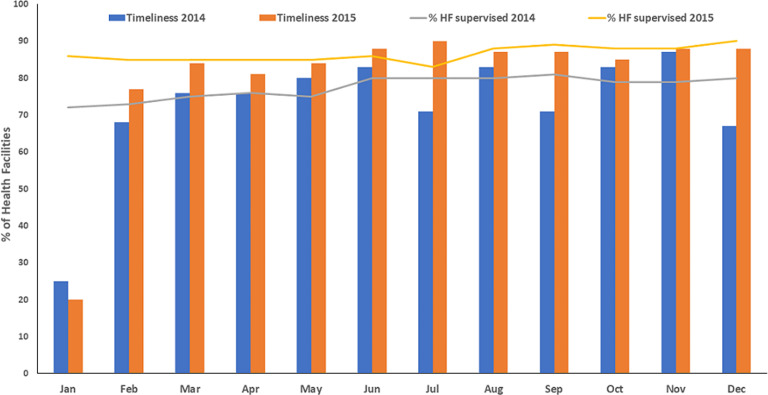
Pimeliness of reporting pre- and post- implementation of the routine immunization module of the district health information system version 2 in supervised health facilities, Kano, 2014 - 2015

The key RI data indicators - completeness and timeliness reporting - were monitored before and after the DHIS2 RI module implementation across 44 LGAs in Kano. They showed some improvement from 2014 to 2015. The number of LGAs with complete HF monthly RI data increased from 30/44(70%) in 2014 to 39/44(87%) in 2015, while LGAs reporting their data in a timely manner increased from 29/44(67%) in the month of December 2014 to 40/44(88%) in December 2015. Supportive supervision and mentoring visits from the state to the LGA and HF levels were not routinely monitored previous to DHIS2 pilot; since the pilot, however, reported supportive supervision visits from LGAs improved from 35 out of 44 LGAs (80%) in December 2014 to 40/44 LGAs (90%) by December 2015 (Figure 2).

**Figure 3:**
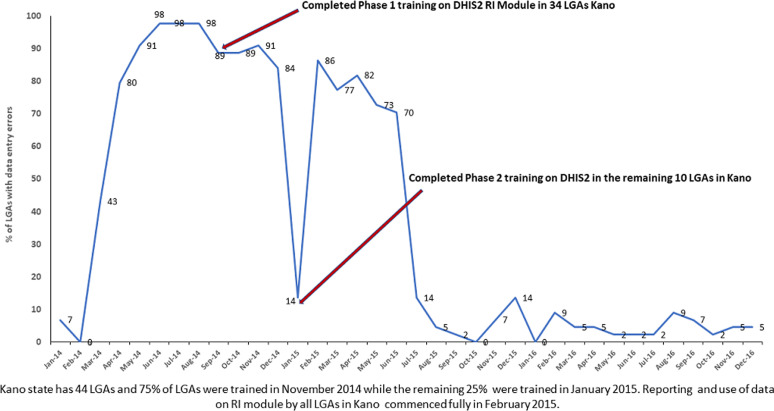
Proportion of LGAs with detected data entry errors pre- and post- implementation of the immunization module of the district health information system version 2, Kano, 2014 - 2016

The review of RI dashboard indicators was one of the activities routinely tracked during the pilot, and the proportion of LGAs that reviewed their dashboard during monthly meetings with HFs increased from 17(40%) out of 44 LGAs in January 2015 to 37(84%) in December 2015. One proxy indicator used for data quality improvement was the reduction in the number of LGAs in Kano with data entry errors/inconsistencies detected on the DHIS2 platform; this number was highly variable but decreased overall from August 2015 through December 2016 (Figure 3).

A total of 12 meetings were held by the DHIS2 core group during the pilot and 176 health facilities are visited per month at 4 per LGA making 14% health facilities in Kano were visited per month during the pilot. Also, telephone calls were made to underperforming health facilities when a visit cannot be conducted. Decisions made based on the data included releasing fund for supportive supervision for LGA officers to visit health facilities, monitoring the roll out of routine inactive polio vaccine (IPV), capacity building activities at all LGA to improve capacity for filling RI data tools and entering data into DHIS2 platform.

## Discussion

The pilot implementation of a DHIS2 RI module in Kano State led to modest improvements in the timeliness and completeness of NHMIS reporting and data quality. The systematic review of indicators by the state immunization officer and follow-up supportive supervisory visits for correction of issues helped to identify week health facilities and LGA personnel for capacity building which may have contributed to the reduction in the number of LGAs with data transfer and entry errors. Overall monitoring by the DHIS2 Core Group and M&E Working Group ensured that problems were being addressed.

These findings are consistent with the experience of other African countries following introduction of district-based data management information system which led to improved routine data reporting. In Uganda, the introduction of a Web-based system facilitated the collection of more accurate RI data to inform planning and decision-making [1]. The implementation of DHIS2 improved data completeness and overall accuracy in South Africa [13].

The degree of improvement of completeness and timeliness of reporting indicators was significant over the one-year period. With more effort, Kano state monthly completeness and timeliness of reporting was at 95% which resulted from continuous implementation of strategies identified during the pilot. A limitation of the study is that the indicators were assessed only for a limited time; however long-term and sustainable increases in reporting and data quality was anticipated.

The implementation of the DHIS2 pilot in Kano presented many challenges: 1) competing activities in the state led to splitting the training into two phases, 2) a limited number of available facilitators led to class sizes of 50-60 trainees instead of the recommended 30 trainees, 3) the time allotted for the hands-on exercises during the training was insufficient, therefore, a follow-up “one-on-one” mentorship was required for trained personnel, 4) poor internet connectivity during and after the training and irregular power supply affected effective and timely input of data into the platform; 50% of LIOs had to travel to the state headquarters on a monthly basis to enter data into the DHIS2 platform and this had implications on data entry and usage of the platform, 5) security challenges in some LGAs affected complete and timely submission of data, and 6) health workers strikes and staff attrition affected reporting.

Despite the challenges faced, a number of successes were noted: 1) full commitment by the state government, 2) full availability of RI data tools in all LGAs and HFs with training of personnel on proper completion, 3) systematic review of the RI dashboard in monthly LGA and state review meetings for intervention, 4) the DHIS2 Core Group, subsequently the M&E Working Group, systematically reviewed RI dashboard indicators allowing for prompt action to address the gaps identified, if not already addressed by LGA personnel, 5) improved reporting rates and accuracy over time, 6) through network mapping, identification of an internet provider that had widespread coverage and creation of a user group to address the challenges of internet connectivity across the LGAs, and 7) improved data quality as demonstrated by declines in administrative vaccination coverage over time from over 100%(?) to values considered to be more realistic by the state (data not presented).

The implementation of DHIS2 pilot in Kano provided several lessons that guided subsequent scale-up to other states in Nigeria: 1) to address the challenge of improper filling of RI data tools, training on these should be held in separate sessions from the DHIS2 training which would allow time for data from the HF forms to be summarized into the new vaccine management monthly summary and NHMIS supplementary forms, 2) revised RI data tools should be distributed to all HFs and old tools destroyed at the time of the training to minimize the possibility of the use of outdated tools, 3) alternative internet sources should be provided at the training venue and the participants should be requested to come with information on available internet providers in the LGAs in order to address the challenge of internet connectivity as session in the training, 4) dedicated internet routers should be provided by the LGA health office with monthly data subscription to provide free internet for LGAs, 5) logistics for cascade trainings should be made available in a timely manner for smooth implementation. 6) funding opportunities should be maximized to ensure transit logistics are available for supportive supervision and mentoring on the utilization of the DHIS2 dashboard.

## Conclusion

The pilot of the DHIS2 RI module in Kano was associated with improvements in the completeness and timeliness of DHIS2 data reporting and in data quality which ultimately provide reliable administrative data for programmatic decision making. The implementation of the DHIS2 RI module pilot was supported by full involvement of the various stakeholders, including government at state and local levels and Polio Eradication Initiative partners. The successes and lessons learnt were applied for scaling up throughout all states in Nigeria.

### What is known about this topic

- Strengthening data management using available structures and existing platform in the system has been identified as a key pathway to improving Primary health care data;
- Having multiple layers and levels of data entry and aggregation including person-to-person reporting is known to provide the system with opportunities for data entry errors and poor data quality;
- The DHIS platform offers the opportunity to integrate all health data sources into a single integrated health management information system that can be accessed real time with a dedicated dashboard that makes available data disaggregated at sub administrative levels for easy management and use.

### What this study adds

- This study was able to showcase how the DHIS 2 platform could be used to effectively to manage RI data through a dedicated and customized dashboard to strengthen RI data management and effectively integrate and institutionalize DHIS2 RI specific module into the routine health management information system;
- Findings from this pilot study was able to showcase the usability of the platform and best practices in the system that guided the scale-up of the DHIS2 RI specific module to the entire country.

## References

[1] Kiberu VM, Matovu JKB, Makumbi F, Kyozira C, Mukooyo E, Wanyenze KR (2014). Strengthening district-based health reporting through the district health management information software system: the Ugandan experience. BMC Med Inform Decis Mak.

[2] Ishijima H, Mapunda M, Mndeme M, Sukums F, Mlay SV (2015). Challenges and opportunities for effective adoption of HRH information systems in developing countries: national rollout of HRHIS and TIIS in Tanzania. Hum Resour Health.

[3] Kihuba E, Gathara D, Mwinga S, Mulaku M, Kosgei R, Mogoa W (2014). Assessing the ability of health information systems in hospitals to support evidence-informed decisions in Kenya. Glob Health Action.

[4] National Primary Health Care Development Agency (2013). National Routine Immunization Strategic Plan 2013-2015.

[5] Braa J, Heywood A, Sahay S (2012). Improving quality and use of data through data-use workshops: Zanzibar, United Republic of Tanzania. Bull World Health Organ.

[6] Ophori EA, Tula MY, Azih AV, Okojie R, Ikpo PE (2014). Current trends of immunization in Nigeria: prospect and challenges. Trop Med Health.

[7] Wonodi C, Stokes-prindle C, Aina M, Oni G, Olukowi T, Pate MA (2012). Landscape analysis of routine immunization in Nigeria.

[8] (2009). The National Strategic Health Development Plan Framework (2009-2015) TWG- NSHDP/ Health Sector Development Team. NCH adopted July 2009.

[9] Burton A, Monasch R, Lautenbach B, Gacic-Dobo M, Neill M, Karimov R (2009). WHO and UNICEF estimates of national infant immunization coverage: methods and processes. Bull World Health Organ.

[10] Daskalakis TG (1992). Improving data collection. NAHAM Manage J.

[11] Asangansi I (2012). Understanding HMIS Implementation in a Developing Country Ministry of Health Context-an Institutional Logics Perspective. Online J Public Health Inform.

[12] National Bureau of Statistics Demographic statistics 2015.

[13] Williamson L, Heywood AB, Williamson L (2001). Developing a district health information system in South Africa: a social process or technical solution?. Stud Health Technol Inform.

